# Facile preparation of amine -functionalized corn husk derived activated carbon for effective removal of selected heavy metals from battery recycling wastewater

**DOI:** 10.1016/j.heliyon.2022.e09516

**Published:** 2022-05-23

**Authors:** Muhammad Salihu Ismail, Muibat Diekola Yahya, Manase Auta, Kehinde Shola Obayomi

**Affiliations:** a Department of Chemical Engineering, School of Infrastructure, Process Engineering and Technology, Federal University of Technology, PMB 65, Minna, Niger State, Nigeria; b Department of Chemical Engineering, Landmark University, PMB 1001, Omu-Aran, Kwara State, Nigeria

**Keywords:** Corn husk activated carbon, Amine functionalization, Heavy metals, Adsorption, Desorption

## Abstract

In this work, an efficient and eco-friendly amine functionalized corn husk derived activated carbon with high adsorption capacity was prepared and utilized for Pb (II), Cu(II) and Ni(II) ions removal from battery recycling wastewater. The developed adsorbent was characterized to determine the surface morphology, elemental composition, surface chemistry and surface area using SEM/EDS, FTIR and BET techniques. The BET surface area of the corn husk (CH) and amine-functionalized corn husk activated carbon (AF-CHAC) was found to be 92.11 and 442.70 m^2^/g, respectively. The effect of adsorption variables which includes temperature, pH, contact time, and adsorbent dosage on uptake behaviour were all examined. Langmuir, Freundlich, Harkin-Jura, Elovich, and D–R isotherm models were fitted to the adsorption data. The adsorption of Pb (II), Cu(II), and Ni (II) ions followed a pseudo-second order kinetic and fit well to the Freundlich isotherm, indicating multi-layer adsorption and chemisorption. The maximum adsorption capacity of Pb(II), Cu(II), and Ni(II) ions, was 2.814, 0.724, and 0.337 mg/g, respectively. According to the thermodynamic parameter values, the adsorption process was spontaneous, exothermic, and physical in nature, with an increase in randomness at the adsorbates-adsorbent interaction. The desorption and reusability experiments revealed that the AF-CHAC has a greater potential as an adsorbent, with a removal efficiency of 99 % after three cycles. Overall, the prepared amine functionalized corn husk derived activated carbon has advantages such as ease of preparation, cost effectiveness, and excellent recyclability, as well as high adsorption capacity, providing a new approach for efficiently treating battery recycling wastewater contaminated with heavy metal ions.

## 1 Introduction

In developing and underdeveloped countries, environmental pollution caused by the continuous release of toxic substances into waters has a result of rapid development of urbanization and industrialization has become a major concern worldwide most especially in developing and undeveloped countries (Obayomi et al., 2021; Lin et al., 2022). Several pollutants, such as industrial dyes, drugs, agrochemicals, and heavy metals, can affect organisms, including humans, directly or indirectly (Obayomi et al., 2022a). Due to their non-biodegradability, toxicity, and persistence, water pollution caused by heavy metal contamination even at trace concentrations, has already been proven to pose a threat to human health and the environment (Bhattacharjee et al., 2021).

The need to mitigate heavy metal pollution from wastewater has necessitated the use of several techniques which includes precipitation (Zhang and Duan, 2020), membrane filtration (Ercarikci and Alanyalioglu, 2021), flocculation (Liu et al., 2021), and adsorption (Chai et al., 2021). Adsorption has proven to be the most preferred removal technique among the techniques mentioned due to its cost effectiveness, fast and highly efficient removal, simple operation, excellent surface area and high efficiency removal (Li et al., 2022).

With the rapid advancement of material science, newer adsorbents with high adsorption capacity, cost effectiveness, eco-friendliness, and selectivity are being reported (Hashem et al., 2021). Several research works have reported the use of different materials beginning with chitosan (Zarghami et al., 2016), clay (Obayomi and Auta, 2019), silica (Radi et al., 2019) zeolites (Cheng et al., 2021), carbon nanotubes (Khan et al., 2021), multiwall carbon nanotubes (Obayomi et al., 2020), metal organic framework (Ahmadijokani et al., 2021), and covalent organic framework (Xiao et al., 2021) amongst others to be utilized as an adsorbent for the treatment of heavy metal pollutants in wastewater. Low sorption and desorption efficiency, unstable chemically at low pH, low selectivity and cost effectiveness are the challenges associated with the aforementioned adsorbents (Obayomi et al., 2022b).

Researchers' recent drive to discover and develop an effective and dependable adsorbent has consistently been a crucial pursuit for it to be commercialized and widely used in industries. Therefore, there is a pressing need to prepare adsorbents with high adsorption capacity, ease of separation, chemical stability, eco-friendliness, and low cost (Yahya et al., 2020a). Activated carbon as an adsorbent has become an efficient and promising materials for the treatment of heavy metal contaminated wastewater as a result of their large surface area, development of abundant internal pore structure, high selectivity, ease of operation (Waly et al., 2021). However, the drawbacks associated with commercialized activated carbon such as its relatively high cost, low adsorption capacity, and low regenerability, among others, have limited its widespread application in wastewater treatment, particularly in developing countries. Considering the aforementioned challenges, several studies have recently focused great attention on developing activated carbon from waste biomass due to its specific properties such as enriched surface functional groups, highly porous structure, large surface area, and relatively cost-effective preparation methods in order to improve economic viability (Egirani et al., 2021). Adsorbent preparation from waste biomass materials is beneficial to waste management and reduces the cost of adsorbent materials. Corn is a staple food that is widely consumed on a global scale, and it also produces a significant amount of waste husk (Mishra et al., 2019). Corn husks are the outer coverings of corn ears that have a high carbon content and can be converted into porous carbon material for wastewater treatment.

Different materials have been reportedly used as supports for activated carbon functionalization; however, due to their low surface area and limited binding sites, most of these supports are often unsuitable for industrial applications (Mosayebi et al., 2020). Thus, finding suitable, efficient, and robust supports is a vital need for the industrialization of this technology and is a hotly debated research topic. As a result, the surface functional groups of activated carbon (hydroxyl and carboxylic acid groups) are chemically modified with chelating groups to improve its adsorption capacity for the removal of toxic heavy metals from wastewater (amine and thiol groups).

In this study, activated carbon from corn husk (CHAC) was created and functionalized with an amine group (AF-CHAC) to create novel supporting materials with a high surface area, high adsorption capacity, and reusability for the effective removal of heavy metals from wastewater. The effect of contact time, adsorbent dosage, temperature, and pH on the adsorption behavior of AF-CHAC under batch conditions were studied. AF-CHAC adsorption kinetics, isotherms, thermodynamics, and regeneration studies were performed to assess the performance of its practical application in the treatment of Pb(II), Cu(II), and Ni(II) ions from wastewater.

## 2 Materials and methods

### 2.1 Materials

Corn husk was sourced from local commercial corn roasters in Minna, Nigeria. Epichlorohydrin (C_3_H_5_ClO), hydrochloric acid (HCl), N, N-dimethylformamide ((CH₃)₂NCH), sodium chloride (NaCl), ethylenediamine (C₂H₄(NH₂)₂), sodium hydroxide (NaOH), and nitric acid (HNO_3_), and of analytical grade were procured from Sigma Aldrich alongside other chemicals and reagents utilized in this study. The battery recycling wastewater was gotten from a battery industry in Nigeria. The proximate analysis of the obtained raw corn husk (CH) was determined using the established experimental procedure described by Mishra et al. (2019).

### 2.2 Activated carbon preparation

The method described by Obayomi et al. (2021) with some modifications was utilized to synthesize activated carbon derived from corn husk activated carbon (CHAC). The corn husk was thoroughly rinsed with distilled water, sun-dried before being oven dried at 110 °C for 6 h, grounded and crushed to a particle size of 250 μm. 200 mL of HNO_3_ (2 % v/v) was added 30 g of sieved corn husk in a 500 mL beaker; then the mixture was stirred for 2h on a magnetic stirrer and kept overnight. After impregnation, the mixture was thoroughly washed with distilled water severally to attain a neutral pH and dried in an oven at 108 °C for 2 h. The dried impregnated corn husk was placed in a crucible and heated at 300 °C for 2 h at a heating rate of 20 °C/min under a purified nitrogen (99.99 %) atmosphere and a flow rate of 100 cm^3^/min. After allowing the synthesized material (CHAC) to cool at room temperature, it was homogenized and stored in an airtight container for functionalization with amine.

### 2.3 Preparation of amine functionalized corn husk activated carbon

The approach described by Peng et al. (2017) was employed with few modifications to prepare amine functionalized corn husk activated carbon (AF-CHAC). Briefly, the intermediate reaction was made by mixing 20 mL epichlorohydrin, 25 mL N, N-dimethylformamide and 11 mL ethylenediamine in a 250 mL round bottom flask at 80 °C on a rotary magnetic stirrer at 100 rpm for 1 h. 25 mL of trimethylamine was added to the solution afterwards and stirred for another 1 h. 20 mL pyridine (as the catalyzer) and 20 of CHAC was added to the mixture and stirred at 90 °C for 2 h. After the reaction was completed, the resultant liquid was filtered and washed with 500 mL of 50 % ethanol and distilled water before being dried at 75 °C for 6 h. The prepared amine functionalized corn husk activated carbon (AF-CHAC) was then stored for further usage.

### 2.4 Characterization of adsorbent

The prepared AF-CHAC point of zero charge was carried out using the experimental method described by Obayomi et al. (2022a). A 50 mL solution of 0.01 M NaCl was prepared and measured into ten different 100 mL conical flasks, and 0.5 g AF-CHAC was added to each flask. 0.1 M HCl and 0.1 M NaOH solutions were used to adjust the pH of each flask to a range of 2–11. The flasks were shaken vigorously for 48 h on a mechanical shaker. Following the completion of the reaction, the mixtures were filtered, and the pH of each flask was measured. The pHZPC value was then calculated by plotting the graph of pH_initial_ versus pH_final_ and finding the point of intersection. The scanning electron micrograph (SEM/EDS, A JSM 4490 JEOL, Japan) was used to determine the surface morphology and elemental analysis of CH and AF-CHAC. The functional groups of the prepared surface materials were studied using Fourier-transform infrared spectra (FTIR, model; NICOLET 6700, Thermo-Fisher Scientific, U.S.A.). The Brunauer Emmett Teller (BET, TristarTM II 3020, Micromeritics, USA) method was used to evaluate the adsorbent pore size, the surface area, and pore volume.

### 2.5 Batch adsorption experiments

In a 250 mL Erlenmeyer flask, experimental batch adsorption studies were carried out using an orbital shaker set to 140 rpm. All experiments were conducted at a temperature of 25 °C. 100 mL aqueous solution of the battery recycling wastewater was measured and transferred to a 250 Erlenmeyer flask, where a predetermined AF-CHAC dosage was added. The solution pH was altered using 0.1 M solutions of HCl and NaOH. After a predetermined time period, the solution was filtered and analyzed to determine the residual concentrations of Pb(II), Cu(II), and Ni(II) ions.

Process optimization was investigated by varying the AF-CHAC dosage (1–7 g/L), pH (2–11), contact time (0–120 min), and temperature (298–328 K). To reduce errors, all experiments were carried out in triplicate. Thereafter, samples were taken at various time interval and filtered through a 45 mm micropore-filter membrane to measure the residual concentration of the metal ions using the Atomic Absorption Spectroscopy (Perkin Elmer, Model A. Analyst 200). The metal ions percentage uptake, amount adsorbed at equilibrium and at different intervals of time, t (mg/g), were calculated using the equations

(1) $$\text{Uptake ​}\left(\text{\%}\right)=\left(\frac{{\text{C}}_{\text{o}}-{\text{C}}_{\text{e}}}{{\text{C}}_{\text{o}}}\right)\times 100$$

(2) $${\text{q}}_{\text{e}}=\frac{\left({\text{C}}_{0}-{\text{C}}_{\text{e}}\right)\text{ ​V}}{\text{W}}$$

(3) $${\text{q}}_{\text{t}}=\frac{\left({\text{C}}_{0}-{\text{C}}_{\text{t}}\right)\text{ ​V}}{\text{W}}$$

Where C_o_, C_e_ and C_t_ are the initial concentrations, equilibrium concentrations and concentrations at different time of the metal ions (mg/L); V is the solution dye volume (L); and W is the adsorbent weight (g). q_t_ and q_e_ represents the amount of metal ions adsorbed at different time interval, t and at equilibrium (mg/g).

### 2.6 Desorption and reusability studies

0.5 g of AF-CHAC was added to a 50 mL solution of the battery recycling wastewater at pH of 8. The AF-CHAC adsorbent was filtered after 2 h, and the metal ion concentration was measured. 0.5 g of metal-loaded AF-CHAC was then collected and agitated with 50 mL of 0.5 M HNO_3_ for 2 h (to remove the metal ions from the adsorbent surface). The AF-CHAC adsorbent was filtered after desorption, dried at 108 °C for another 2 h, and regenerated with 50 mL of 0.5 M NaOH solution to restore the negative charge and amino groups. Following that, the regenerated adsorbent was filtered, dried, and reused up to five times.

## 3 Results and discussion

### 3.1 Proximate analysis

Prior to carbonization, activation and functionalization, the proximate analysis of raw corn husk was determined, and the result was presented in Table 1. The low moisture content of 12.50 % can be attributed to the fact that corn husk was first sun dried before being dried in an oven. The presence of organic matter in the corn husk, which can be attributed to where the material was sourced, resulted in a volatile content of 20.84 %. The material ash content was found to be 11.67 %, indicating that the biomass contained inorganic and non-combustible materials. The high fixed carbon content of 54.99 % represents the amount of carbon present in the corn husk after removing the moisture, ash, and volatile contents, recommending the material as excellent precursor for the synthesis of activated carbon. This finding was in close agreement with the result obtained by Pallarés et al. (2018).

**Table 1:** Proximate analysis of corn husk.

S/n	Weight (%)
1	Moisture Content 12.50
2	Ash content 11.67
3	Volatile content 20.84
4	Fixed carbon 54.99

### 3.2 Adsorbent characterization

#### 3.2.1 Point of zero charge (pHZPC)

The pHZPC of an adsorbent is the pH value at which the adsorbent's surface is free of charge, as well as the pH value that determines whether the adsorbent is basic or acidic. The adsorbent surface will be positively charged if the pH is less than pHZPC; negatively charged if the pH is greater than pHZPC. The AF-CHAC point of zero charge was estimated to be 6.8.

#### 3.2.2 Surface chemistry analysis

**Figure 1:**
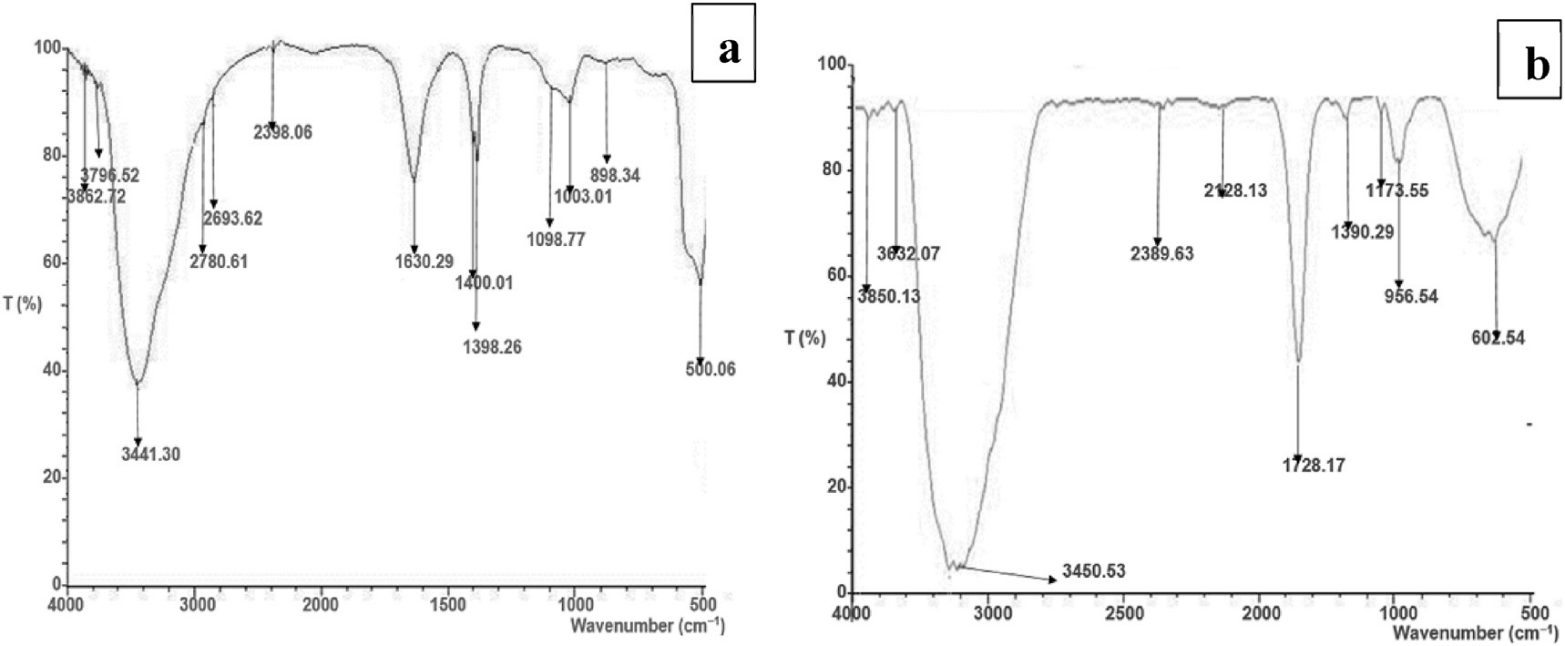
FT-IR spectra analysis of (a) CH, and (b) AF-CHAC.

Figure 1(a) shows the FT-IR spectra of corn husk with an intense peak at 3862.72 cm^−1^ which can be attributed to the stretching of O–H group due to inter and intra molecular hydrogen bonding such as alcohols or phenols (Debnath et al., 2020). The peak observed at 3796.52 cm^−1^ can be associated with C–H asymmetric stretching vibration of the CH_2_ group (Pachathu et al., 2016). The C–H bond of methoxy groups was noticed at a broad absorption peak at 3441.30 cm^−1^ (El-Sakhawi et al., 2018). The absorption band at 2780.61 cm^−1^ was attributed to the C–H associated with alkynes and aromatics (Ali et al., 2019). An absorption band at 2398.06 cm^−1^ was attributed to the C=O stretching vibrations of carboxylic while the steep peaks observed around 1630.29 to 1398.26 cm^−1^ corresponded to the C–C stretching, and C–O asymmetric stretch which might be attributed to the presence of an aromatic or olefinic compounds (Lin et al., 2019). The C–Br Stretching vibration and C–O stretching of alcohol or carboxylic acid were recorded at peaks ranging from 1003.01 to 898.34 cm^−1^ (Indha et al., 2016). Figure 1(b) shows the FT-IR spectrum of the amine-modified adsorbent, which shows peaks at 3850.13, 3632.07, 3450.53, 2389.63, 2128.13, 956.10, and 602.54 cm^−1^, which are attributed to the O–H stretching vibration, C–H asymmetric stretching vibration of the CH_2_ group connection, C–H stretching vibration, C=O stretching vibrations, and C–Br stretching vibrations, respectively. The N–H bending vibration and C=O stretching of aldehydes and ketones, which results from the functionalization of the adsorbent, were linked to the occurrence of emerging intense peaks at 1390.27 and 1173.55 cm^−1^. This finding is in agreement with Rashid et al. (2019) who studied the utilization of naturally functionalized pumpkin peels for methylene blue decolorization.

#### 3.2.3 Surface morphology

**Figure 2:**
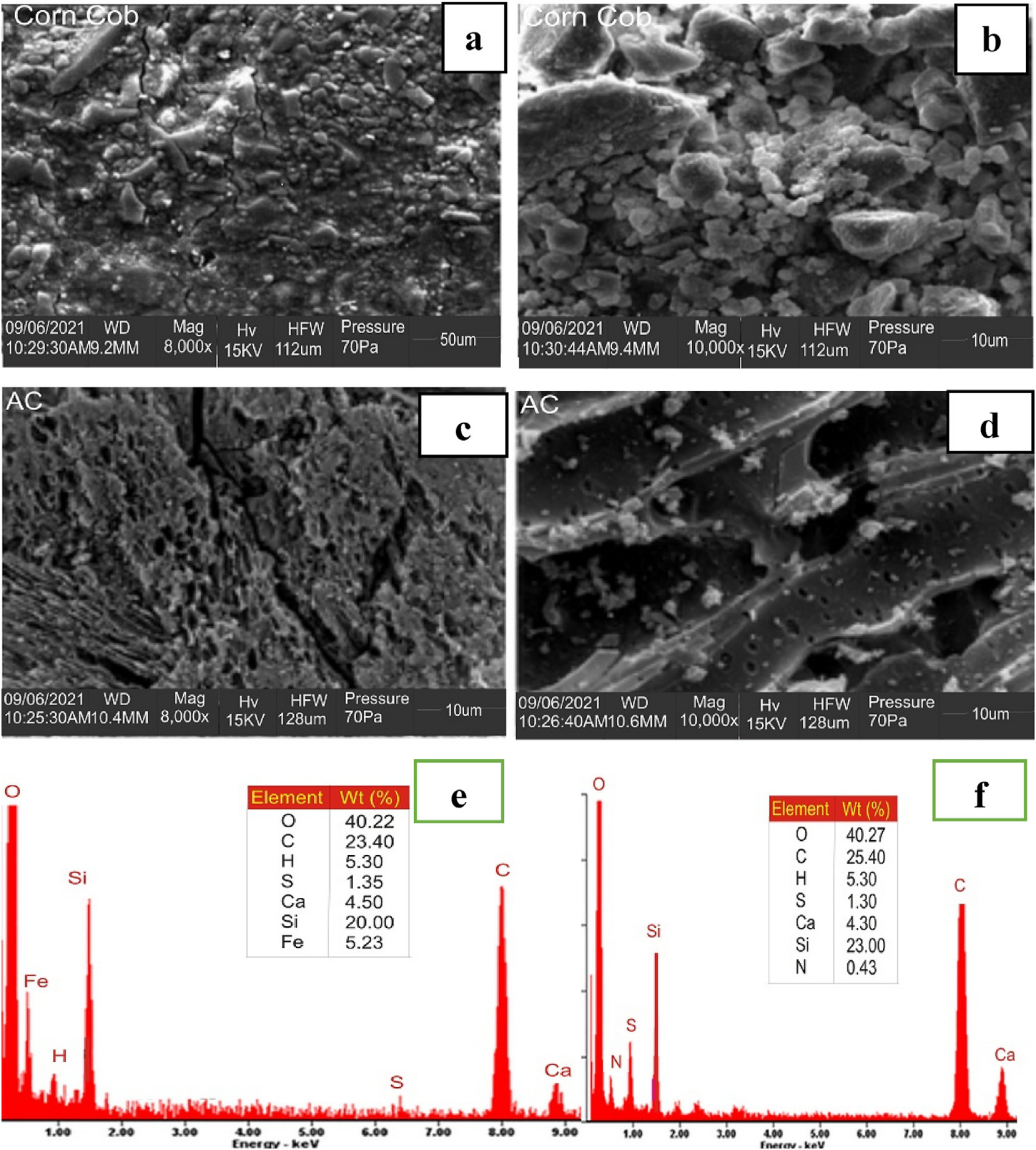
SEM micrograph and EDS analysis for (a, b, & d) CH, and (c, d,& f) AF-CHAC.

The micrograph and elemental analysis of CH and AF-CHAC were determined at various magnifications, and the results are shown in Figure 2. The SEM images of CH as shown in Figure 2(a, b) demonstrate dense, densely packed, uneven, rough pores and cavities. The SEM images of AF-CHAC after carbonization, chemical activation, and amine functionalization shown in Figure 2 (c, d) show the presence of more pores on the surface of the adsorbent material due to the removal of lignin, hemicellulose, and cellulose, as well as the addition of some functional groups, allowing for the easy transfer of adsorbates into the inner surface of the adsorbent material (Mandal et al., 2021). The EDS analysis of CH as shown in Figure 2(e), reveals the presence of elements such as O, C, H, S, Ca, Si, and Fe, whereas the elements present in AF-CHAC as depicted Figure 2(f), include O, C, H, S, Ca, Si, and N, with a higher weight percent of C and O when compared to CH. The amination reaction is responsible for the replacement of Fe with nitrogen-containing functional groups (Mishra et al., 2019).

#### 3.2.4 BET analysis

BET analysis of CH and AF-CHAC as well as comparisons with other adsorbents was presented in Table 2. The result revealed that the BET surface areas of CH and AF-CHAC was found to be 92.11 and 442.70 m^2^/g, respectively. The increase in the BET surface area of AF-CHAC can be attributed to the chemical activation and amine functionalization of the CH material.

**Table 2:** BET surface area of CH and AF-CHAC in comparison with other adsorbents.

Adsorbent	Surface area (m^2^/g)	References
Raw Corn husk	92.11	This study
F-CHAC	442.70	This study
Pumpkin peels	360	Rashid et al. (2019)
Magnetic functionalized activated carbon	48.9	Fu et al. (2016)
Magnetic nanoparticles	432	Fatehi et al. (2017)

### 3.3 Adsorption studies

#### 3.3.1 Effect of solution pH

**Figure 3:**
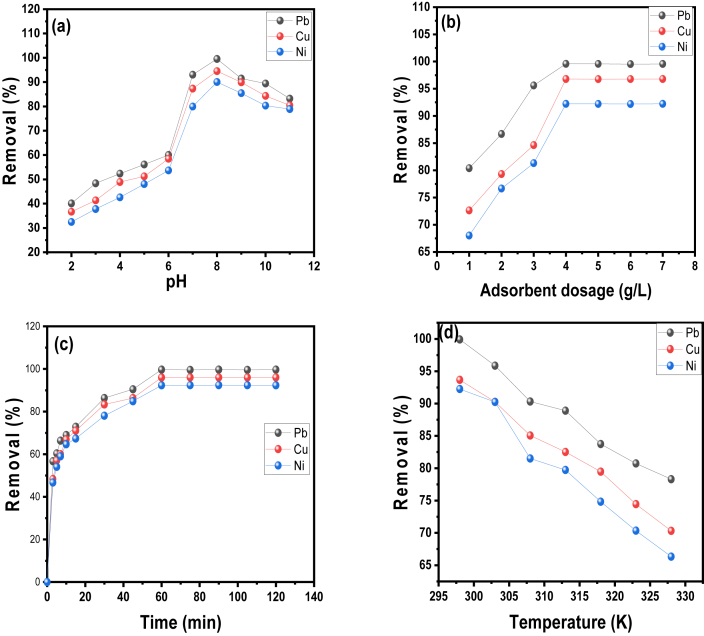
Effect of adsorption parameters (a) pH (2–11) (b) contact time (0–120 min) (c) Adsorbent dosage (1–7 g/L), and (d) temperature (298–328 K) on the removal of Pb(II), Cu(II), and Ni(II) ions.

The pH value is one of the most important parameters that governs the adsorption process. This is because the pH value affects the properties of the adsorbate as well as the surface adsorption sites (Fu et al., 2016). As shown in Figure 3(a), the percentage removal of Pb(II), Cu(II), and Ni(II) ions was observed to have increased as the pH value increased from 2-8, with further pH increases beyond this point (>8) resulting in no significant metal ion uptake. As the pH value was varied from 2 to 8, the percentage removal of the heavy metal ions increased from 40.08 to 99.50 %, 36.66–94.51 %, and 32.45–90.04 %, respectively. The pHpzc of AF-CHAC was calculated to be 6.8, and at pH values above the pHpzc value, the surface of the AF-CHAC becomes negatively charged due to deprotonation (presence of OH^−^), resulting in electrostatic interaction of the positively charge metal ions (Yahya et al., 2020b). The presence of hydronium ions (protonation) on the surface of the adsorbent (pH value is below the pHpzc value) competing with the positively charged Pb(II), Cu(II), and Ni(II) ions resulted in removal decreased due to electrostatic repulsion (Khan et al., 2020). Finally, increasing the pH value (>8) resulted in a decrease in the percentage removal of metal ions, which could be attributed to the hydroxide formation of Pb(II), Cu(II), and Ni(II) ions due to chemical precipitation (Peng et al., 2017).

#### 3.3.2 Effect of adsorbent dosage

The effect of AF-CHAC dosage on the percentage removal of Pb(II), Cu(II), and Ni(II) ions was investigated by varying the adsorbent dosage from 1-7 g/L. It was clear from the results shown in Figure 3(b) that increasing AF-CHAC resulted in increased heavy metal ions removal. The removal efficiencies of Pb(II), Cu(II), and Ni(II) ions increased from 80.39 to 99.58 %, 72.64–93.79 %, and 68.02–89.63 %, respectively, at a dosage of 1–4 g/L. The availability of more adsorbent and adsorption sites for the adsorbate's molecules could explain the likely percentage removal increase. The percentage removal of the heavy metal ions is in the following order: Pb > Cu > Ni. Pb (II) ions have the highest percentage removal due to their high electronegativity of 2.33, followed by Ni ions with 1.91 and Cu with 1.90. The higher the electronegativity, the faster the diffusion and removal efficiency (Egbosiuba et al., 2020; Mustapha et al., 2020). Finally, the optimal AF-CHAC dosage for the removal of Pb(II), Cu(II), and Ni(II) ions was 4 g/L. Therefore, it was observed that increasing the adsorbent dosage (>4 g/L) resulted in no higher heavy metal ions uptake, suggesting that the optimum ratio of adsorbent to adsorbate dosage was reached and all of the active sites on the adsorbent dosage were occupied (Obayomi et al., 2022a).

#### 3.3.3 Effect of contact time

The effect of contact time which is an important component in determining the removal efficiency was studied on the AF-CHAC performance for the removal of Pb(II), Cu(II), and Ni(II) ions from battery recycling process wastewater. The removal efficiency was investigated in the range of 0–120 min, pH 8, at 298 K and adsorbent dosage of 4 g/L. As depicted in Figure 3(c), it was observed that the percentage removal of Pb(II), Cu(II), and Ni(II) ions increased significantly with contact time increased. The percentage removal of the heavy metal ions was observed within the first 5 min to be rapid due to the availability of more vacant sites and active functional group on the surface of AF-CHAC. The maximum percentage removal for Pb(II), Cu(II), and Ni(II) ions was 99.66, 96.01, and 92.36 %, respectively and this was observed at an equilibrium time of 60 min. Furthermore, increasing the contact time beyond this point resulted in a percentage removal decrease due to blockage or saturation of the adsorbent active sites and the weakening of the binding force as the sorbate concentration decreases (Mustapha et al., 2020). Similar observation was reported by Fatehi et al. (2017) who functionalized magnetic nanoparticles supported on activated carbon for removal of Pb(II) and Cr(VI) ions from Saline Solutions.

#### 3.3.4 Effect of temperature

The temperature effect on the percentage uptake of Pb (II), Cu(II), and Ni(II) ions onto AF-CHAC was investigated using a temperature range of 298–338 K, a pH of 8, a contact time of 60 min, and an adsorbent dosage of 4 g/L. The percentage uptake of the heavy metal ions decreased with increasing temperature, suggesting an exothermic nature of the adsorption process. The highest percentage removal of Pb(II), Cu(II) and Ni(II) at 298 K was found to be 99.91, 93.66 and 92.24 %. as shown in Figure 3(d).

### 3.4 Adsorption isotherm

The equilibrium adsorption of Pb, Cu, and Ni ions onto AF-CHAC was used to assess th adsorption isotherm that explains the distribution of adsorbate molecules across the adsorbent surface. Langmuir, Freundlich, Harkin-Jura, Elovich, and Dubinin–Radushkevich (D–R) isotherm models were chosen, and their linear form of equation is presented in Table 3. The Langmuir isotherm is based on the assumption of a homogeneous surface with equivalent sorption energies and no interactions between adsorbed species. The Freundlich isotherm assumption is used to describe not only monolayer coverage but also multilayer adsorption. The Dubinin-Radushkevich (D-R) isotherm model is an empirical adsorption model for expressing adsorption mechanisms on heterogeneous surfaces with Gaussian energy distribution. The Elovich model, which is based on a kinetic principle, assumes that adsorption sites increase exponentially with adsorption, implying multilayer adsorption. The Harkin-Jura isotherm assumes multilayer adsorption on the adsorbent surface with heterogeneous pore distribution.

**Table 3:** Isotherm model equations.

Models	Equation	Plot	Parameters	References
Langmuir	$\frac{{\text{C}}_{\text{e}}}{{\text{q}}_{\text{e}}}$_=_$\frac{1}{{\text{q}}_{\text{m}}{\text{K}}_{\text{L}}}$_+_$\frac{{\text{C}}_{\text{e}}}{{\text{q}}_{\text{m}}}$	$\frac{{\text{C}}_{\text{e}}}{{\text{q}}_{\text{e}}}{\text{vs ​c}}_{\text{e}}$	${\text{q}}_{\text{m}},{\text{ ​K}}_{\text{L}}$	Langmuir (1916)
Freundlich	${\text{logq}}_{\text{e}}={\text{ ​logK}}_{\text{F}}$ +$\frac{1}{\text{n}}{\text{logC}}_{\text{e}}$	$\log{\text{q}}_{\text{e}}\text{ ​vs}\log{\text{C}}_{\text{e}}$	${\text{K}}_{\text{F}},\frac{1}{\text{n}}$	Freundlich (1906)
Temkin	${\text{q}}_{\text{e ​}=}$ B $\ln{\text{k}}_{\text{T}}$ + $\text{B}\ln{\text{C}}_{\text{e}}$	${\text{q}}_{\text{e}}\text{ ​vs}\ln{\text{C}}_{\text{e}}$	${\text{A}}_{\text{T}},\text{ ​B}$	Tempkin and Pyzhev (1904)
Dubinin-Radushkevich (D-R)	$\ln\left({\text{q}}_{\text{e}}\right)=\ln\left({\text{q}}_{\text{s}}\right)-\text{β}{\text{ε}}^{2}$ E = $\frac{1}{\sqrt{2\text{β}}}$	$\ln{\text{q}}_{\text{e}}\text{ ​vs ​}{\text{ε}}^{2}$	${\text{q}}_{\text{s}},\text{ ​β},\text{ ​E}$	Dubinin and Radushkevich (1947)
Elovich	$\ln\frac{{\text{q}}_{\text{e}}}{{\text{C}}_{\text{e}}}=\ln{\text{K}}_{\text{e}}{\text{q}}_{\text{m}}-\frac{{\text{q}}_{\text{e}}}{{\text{q}}_{\text{m}}}$	$\ln\left(\frac{{\text{q}}_{\text{e}}}{{\text{C}}_{\text{e}}}\right){\text{ ​vs ​q}}_{\text{e}}$	${\text{K}}_{\text{e}},{\text{ ​q}}_{\text{m}},$	Gubernak et al. (2003)
Harkin-Jura	$\frac{1}{{{\text{q}}_{\text{e}}}^{2}}=\frac{\text{B}}{\text{A}}-\left(\frac{1}{\text{A}}\right)\log{\text{C}}_{\text{e}}$	$\frac{1}{{{\text{q}}_{\text{e}}}^{2}}\text{ ​vs ​}\log{\text{C}}_{\text{e}}$	B, A	Foo and Hameed (2010)

Where ${\text{q}}_{\text{m}}$ is the maximum adsorption capacity of metal ions (mg/g); ${\text{K}}_{\text{L}}$ is the Langmuir isotherm constant (L/g); ${\text{K}}_{\text{f}}$ is Freundlich constant related to adsorption capacity, n is the adsorption intensity; ${\text{q}}_{\text{s}}$ is the theoretical isotherm saturation capacity (mg/g); β is Dubinin–Radushkevich isotherm constant (mol^2^/kJ^2^);  ​ ​E is the mean free energy (kJ/mol); R is the universal gas constant (8.314 J/mol K); T is the temperature in Kelvin (K); B and A are Harkin-Jura constants; and ${\text{K}}_{\text{e}}$ is the Elovich constant. The sum of square error, as given by the equation, was used to predict the suitability and fitness of the adsorption isotherm and kinetic model to the adsorption process in comparison to the correlation coefficient (R^2^)

(4) $$\text{SSE ​}=\overset{\text{N}}{\underset{\text{i}=1}{\sum }}{\left({\text{q}}_{\text{i},\text{cal}}-{\text{q}}_{\text{i},\text{exp}}\right)}^{2}$$

Where N is the experimental sample number; ${\text{q}}_{\text{i},\text{exp}}$ is the adsorption capacity obtained from experimental results at equilibrium (mg/g); and ${\text{q}}_{\text{i},\text{cal}}$ is the calculated adsorption capacity obtained from the models at equilibrium (mg/g).

Based on the highest R^2^ and smallest SSE values obtained, the results showed that the adsorption of Pb, Cu, and Ni ions onto AF-CHAC was best fitted to the Freundlich model, followed by the Langmuir, Elovich, D-R, and Harkin-Jura models, as shown in Table 4. The fitness of the Freundlich model to the adsorption process suggests that multilayer adsorption occurs (surface heterogeneity) (Karnib et al., 2014). The values of 1/n between 0 and 1 indicate that the adsorption process is favorable with the surface becoming more heterogeneous as the values approach zero. According to the Langmuir equation, maximum adsorption capacities calculated for Pb(II), Cu(II) and Ni(II) was 28.137, 17.236, and 10.369 mg/g, respectively. The D-R model calculated adsorption energy (E) values of 1.290 kJ/mol for Pb(II), 0.707 kJ/mol for Cu(II), and 0.408 kJ/mol for Ni(II), suggested that the heavy metal ions adsorption onto AF-CHAC was physisorption (Bassam et al., 2021). Table 5 compares the maximum adsorption capacity obtained with that of other adsorbents from the literature.

**Table 4:** Adsorption isotherm parameters.

Isotherm	Parameter	Pb(II)	Cu(II)	Ni(II)
Langmuir	${\text{q}}_{\text{m}}$	28.137	17.236	10.369
	${\text{Q}}_{\text{e}}$	2.2580	1.4920	1.0650
	${\text{K}}_{\text{L}}$	0.5392	0.1526	0.1104
	${\text{R}}^{2}$	0.9756	0.9616	0.9490
	SSE	0.31	0.59	0.53
Freundlich	${\text{K}}_{\text{F}}$	1.7968	1.0321	0.6978
	${\text{Q}}_{\text{e}}$	2.2580	1.4920	1.0650
	1/n	0.3139	0.3745	0.5287
	${\text{R}}^{2}$	0.9943	0.9916	0.9966
	SSE	0.2127	0.2115	0.1348
Harkin-Jura	B	1.6874	1.6806	1.6623
	A	5.988	2.7655	1.1429
	${\text{q}}_{\text{e}}$	2.2580	1.4920	1.0650
	R^2^	0.8058	0.7109	0.8135
	SSE	13.91	11.6218	9.0607
Elovich	${\text{K}}_{\text{e}}$	2.3535	1.2054	1.2399
	${\text{q}}_{\text{m}}$	1.6984	1.5929	2.9069
	K_e_	2.2580	1.4920	1.0650
	R^2^	0.9466	0.9221	0.9252
	SSE	2.313	3.0101	4.3925
D-R	β	3.0 × 10^−7^	1.0 × 10^−6^	3.0 × 10^−6^
	E	1.290	0.707	0.408
	${\text{q}}_{\text{e}}$	2.2580	1.4920	1.0650
	${\text{q}}_{\text{m}}$	4.4777	3.4573	2.2922
	${\text{R}}^{2}$	0.9087	0.8849	0.8754
	SSE	4.9371	3.8624	6.5060

**Table 5:** Comparison of F-CHAC's adsorption capacity with other adsorbents.

Adsorbent	Adsorption capacity (mg/g)	Metal ion	Experimental condition	Reference
Cobalt ferrite-supported activated carbon	6.27 23.6	lead and chromium ions from tannery wastewater	pH:5, contact time:80 min, dosage:0.8 g and temperature: 333K	Yahya et al. (2020c)
magnetic activated carbon incorporated with amino groups	104.20	Pb(II)	Dosage 1.0 g/L, pH 2	Fu et al. (2016)
Magnetic iron oxide (Fe3O4) nanoparticles from tea waste	4.81	Arsenic	Contact time 30 min, pH 6, Dosage 3 g/L, Temperature 30 °C	Lunge et al. (2014)
Magnetic Activated Carbon Derived from biomass (Coconut shell) Waste	3.23	Toxic dyes	Contact time 2 h, Dosage 2 g/L, pH 6.1, Temperature 25 °C	Cazetta et al. (2016)
Functionalized corn husk derived Activated carbon	7.95 6.08 4.99	Pb Cu Ni	Contact time 125 min, Dosage 0.5 g, Temperature 25 °C, pH 8	This work

### 3.5 Adsorption kinetics

The adsorption kinetics model can be used to determine the possibility of metal ion adsorption as well as the rate controlling steps. The adsorption kinetic models of pseudo-first order, pseudo-second order, and intraparticle diffusion equations as presented in Table 6 were used to investigate the mechanism of metal ion adsorption onto AF-CHAC.Where k_1_ is the pseudo-first-rate constant (min^−1^), k_2_ is the pseudo-second order rate constant (g/mgmin), kp is the Webber-Morris intraparticle diffusion rate constant (mg/g.min^1/2^), K_F_ (min^−1^) is the liquid film diffusion rate constant, C is the rate constant diffusion.

**Table 6:** Kinetic model equations.

Kinetic models	Linear form	Plot	Parameters	Reference
Pseudo-first order	$\log({\text{q}}_{\text{e ​}-}{\text{q}}_{\text{t}}$) = $\log{\text{q}}_{\text{e ​}-}\frac{{\text{k}}_{1}\text{t}}{2.303}$	$\log({\text{q}}_{\text{e ​}-}{\text{q}}_{\text{t}}$) vs t	${\text{q}}_{\text{e},\text{ ​cal}},{\text{ ​k}}_{1}$	Lagergren and Svenska et al. (1898)
Pseudo-second- order	$\frac{\text{t}}{{\text{q}}_{\text{t}}}$ = $\frac{\text{t}}{{\text{q}}_{\text{e}}}+\frac{1}{{\text{k}}_{2}{\text{q}}_{\text{e}}^{2}}$	$\frac{\text{t}}{{\text{q}}_{\text{t}}}$ vs t	${\text{q}}_{\text{e},\text{ ​cal}},{\text{ ​k}}_{2}$	Ho and McKay (1999)
Webber-Morris intra-particle diffusion	${\text{q}}_{\text{t}}={\text{k}}_{\text{p}}{\text{t}}^{0.5}+\text{ ​C}$	${\text{q}}_{\text{t}}$ vs ${\text{t}}^{0.5}$	${\text{k}}_{\text{p}},\text{ ​C}$	Weber and Morris (1963)

Table 7 summarizes the parameters obtained for pseudo-first order, pseudo-second order and intraparticle diffusion models. In comparison of pseudo-first and pseudo-second kinetic models based on the high R^2^ and lower SSE values, it was seen that the pseudo second-order model best described heavy metal ions adsorption onto AF-CHAC. Furthermore, when compared to the pseudo-first order, the pseudo-second order model calculated adsorption capacities were found to be closer to the experimental adsorption capacities for all metal ions adsorbed. Based on the pseudo second-order model assumption, it is possible to conclude that chemisorption is the primary adsorption mechanism for Pb(II), Cu(II), and Ni(II) ions sorption by AF-CHAC (Ghosal and Gupta, 2017; Shahrokhi-Shahraki et al., 2021). Describing the adsorption steps of metal ions, the intra-particle diffusion model was fitted to the adsorption data. It was observed from the result that the adsorption curves exhibited multi-linear plots with two steps (Figure not shown). The steeper slope of the first liner step is caused by external diffusion, which results in faster mass transfer through the boundary layer, whereas the lower slope of the second liner step is caused by internal diffusion, which demonstrates the slow rate of diffusion inside sorbent micropores. In all adsorption cases, the failure of the intercept to pass through the origin can be attributed to different rates of mass transfer in the primary and secondary steps of adsorption, as well as the presence of an initial boundary layer resistance (Xia et al., 2019; Pan et al., 2020).

**Table 7:** Adsorption kinetic model parameters for Pb, Cu and Ni ions.

First-order	$k_{1}$	$q_{e\;theo}$	$q_{e\;exp}$	$R^{2}$	SSE
Pb(II)	0.002	5.2735	6.422	0.727	1.32
Cu(II)	0.004	3.0063	4.844	0.718	3.38
Ni(II)	0.007	1.9073	4.393	0.825	6.18
**Second-order**	$k_{2}$	$q_{e\;theo}$	$q_{e\;exp}$	$R^{2}$	**SSE**
Pb(II)	0.0065	6.826	6.422	0.9908	0.16
Cu(II)	0.0035	5.173	4.844	0.9453	0.11
Ni(II)	0.0028	3.368	4.393	0.9812	1.05
**Intra-particle diffusion**	**K**_**i**_	$q_{e\;theo}$	$q_{e\;exp}$	$R^{2}$	**SSE**
Pb(II)	0.3197	3.1954	6.422	0.8317	10.41
Cu(II)	0.2405	2.2159	4.844	0.9195	6.91
Ni(II)	0.3203	0.8183	4.393	0.9867	12.78

### 3.6 Adsorption thermodynamics

The temperature effect on the uptake of metal ions onto AF-CHAC, feasibility, spontaneity, and the nature of metal ions-AF-CHAC interactions were evaluated using the following relations;

(5) $$\Delta \text{G}=-\text{RT}\ln{\text{K}}_{0}$$

(6) ΔG=ΔH−TΔS

(7) $${\text{K}}_{0}=\frac{{\text{q}}_{\text{e}}}{{\text{C}}_{\text{e}}}$$

Where ${\text{K}}_{0}$ is related to thermodynamic equilibrium constant, T is the absolute temperature (K) and R is the gas constant (8.314 J/mol K). The ΔS ​and ​ΔH values were evaluated using the equation.

(8) $$\ln{\text{K}}_{0}=\frac{\Delta \text{S ​}}{\text{R}}-\frac{\Delta \text{H}}{\text{RT}}$$

Table 8 summarizes the evaluated thermodynamic parameters for the uptake of Pb, Cu, and Ni ions onto AF-CHAC. A negative enthalpy (Δ H) value indicates that the adsorption of metal ions onto AF-CHAC is exothermic in nature. Negative Gibb's free energy (Δ G) value for all metal ions adsorbed indicate that the adsorption process was spontaneous and favorable of the heavy metals by F-CHAC. The positive entropy (Δ S) value suggests that randomness increased at the adsorbent-adsorbates interface during the adsorption process.

**Table 8:** AF-CHAC reusability test after five cycles.

No. of recycles	Adsorption capacity Q_m_			% Desorption	%Re
	Pb (mg/g)	Cu (mg/g)	Ni (mg/g)		
1	6.1995	4.1515	3.3485	33.32	100
2	6.1585	4.1505	3.3155	33.15	99.33
3	6.1545	4.147	3.3005	32.97	99.27
4	5.371	3.634	3.1595	28.00	86.63
5	4.349	3.199	2.899	25.25	70.15

### 3.7 Desorption and reusability study

Table 8 shows the results of the regeneration study experiment for AF-CHAC, as well as the adsorption capacity of the adsorbent after each cycle. The results show that the AF-CHAC has high adsorption capacities for Pb, Cu, and Ni ions after the first three repeated cycles with near 100% adsorbent regeneration; however, this gradually decreases after the third run as the desorption efficiency decreases from 33% to 25%. This further implies the collapse of the inner and outer pores, or the gradual blockage of the active sites, which leads to a change in the structural composition of the adsorbent as a result of extensive exposure to HCl during the desorption recycle process (Gautam et al., 2014).

## 4 Conclusions

This study reported on the use of amine functionalized corn husk activated carbon (AF-CHAC) for heavy metal removal from local battery recycling wastewater. The incorporation of nitrogen-containing groups into the structure of corn husk activated carbon (CH) confirms the amine functionalization of the corn husk derived adsorbent (AF-CHAC). The surface morphology, surface chemistry, and surface area of the prepared material were determined using scanning electron microscopy (SEM), fourier transform infrared spectroscopy (FTIR), and Brunauer Emmett Teller (BET). The BET surface area of CH and AF-CHAC was determined to be 92.11 and 442.70 m^2^/g, respectively. At an optimum adsorbent dosage of 3 g, pH of 8, temperature of 45 °C, and contact time of 125 min, the highest percentage removal of Pb (II), Cu(II), and Ni(II) ions was calculated to be 99.66, 96.01, and 92.36 %, respectively. The adsorption process of metal ions uptake onto AF-CHAC was best described by Freundlich isotherms, suggesting a multilayer adsorption. The adsorption process followed a second order kinetic model, confirming the role of chemisorption in the adsorption process. According to the thermodynamic parameters, the adsorption process was spontaneous, favorable, exothermic, and physical in nature. The AF-CHAC reusability study revealed that there was no significant loss of adsorption capacity after three cycles of reuse. The findings revealed that AF-CHAC could be successfully used as a low-cost and environmentally friendly adsorbent material for the treatment of heavy metals in wastewater.

## Declarations

### Author contribution statement

Muhammad Salihu Ismail: Performed the experiments; Analyzed and interpreted the data; Contributed reagents, materials, analysis tools or data; Wrote the paper.

Muibat Diekola Yahya & Manase Auta: Conceived and designed the experiments.

Kehinde Shola Obayomi: Analyzed and interpreted the data; Wrote the paper.

### Funding statement

This research did not receive any specific grant from funding agencies in the public, commercial, or not-for-profit sectors.

### Data availability statement

Data will be made available on request.

### Declaration of interests statement

The authors declare no conflict of interest.

### Additional information

No additional information is available for this paper.
